# Supplementary material for: R2R - software to speed the depiction of aesthetic consensus RNA secondary structures
Source: BMC Bioinformatics. 2011 Jan 4;12:3. doi: 10.1186/1471-2105-12-3 (PMC3023696; doi:10.1186/1471-2105-12-3)

demo-multistem-manA

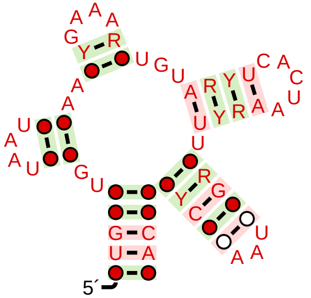

demo-multistem-manA solver=1

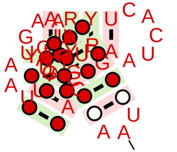

note: R2R cannot find a feasible solution for this problem using the multistem_junction_circular_solver command. However, it can with (1) a user-input optimization starting point, (2) with the try_harder directive or (3) with the multistem_junction_bulgecircley_solver command, as shown in other drawings here.

demo-multistem-manA solver=1explicitinitial

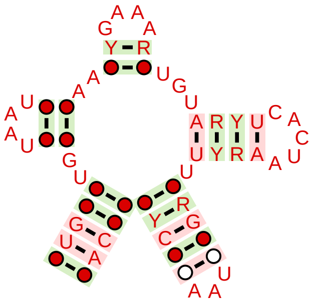

demo-multistem-manA solver=1tryharder

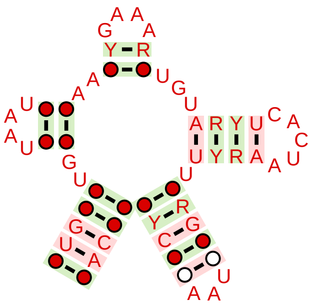

demo-multistem-manA solver=2

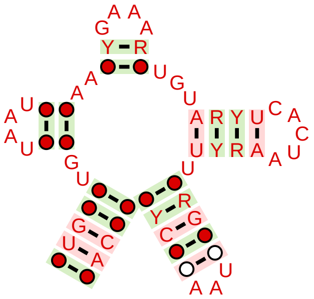

demo-multistem-manA solver=3

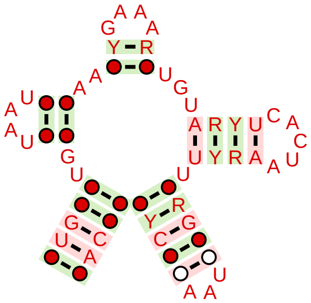

demo-multistem-manA solver=3 annot=1

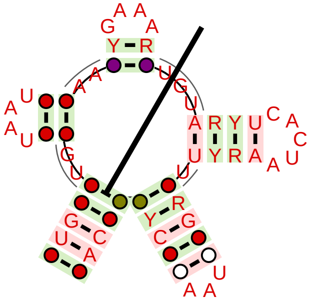

demo-multistem-manA solver=3 annot=1 fewiters=1

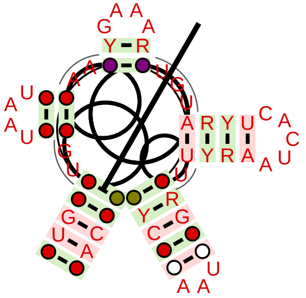

Supplement: Additional file 1 — Source code and example input and output files. C++ and Perl source code is provided. Installation and usage instructions are given in the user manual (Additional file 2), which is also a part of this archive. Example input files for R2R are included in the "demo" subdirectory of this archive, and R2R's raw output on these examples in PDF and SVG format is supplied in the "demo/output-pdf" and "demo/output-svg" subdirectories. Files can be retrieved from the tgz archive file using programs such as WinZip (Windows), StuffIt Expander (Mac) or the tar/gzip commands (UNIX). [file 1471-2105-12-3-S1.TGZ › R2R-1.0.1/demo/output-pdf/demo-multistem-manA.pdf]
